# Plasmonic enhanced OLED efficiency upon silver-polyoxometalate core-shell nanoparticle integration into the hole injection/transport layer

**DOI:** 10.1038/s41598-024-79977-w

**Published:** 2024-11-21

**Authors:** Zoi Georgiopoulou, Apostolis Verykios, Anastasia Soultati, Alexander Chroneos, Anastasia Hiskia, Konstantinos Aidinis, Panagiotis N. Skandamis, Antonia S. Gounadaki, Ioannis Karatasios, Theodoros M. Triantis, Panagiotis Argitis, Leonidas C. Palilis, Maria Vasilopoulou

**Affiliations:** 1 Institute of Nanoscience and Nanotechnology, National Center for Scientific Research ‘Demokritos’, AgiaParaskevi 15341, Athens, Greece; 2 Solid State Physics Section, Department of Physics, National and Kapodistrian University of Athens, Panepistimioupolis, Athens, 15784 Zografos Greece; 3 Department of Electrical and Computer Engineering, University of Thessaly, Volos, 38221 Greece; 4 Department of Materials, Imperial College, London, SW7 2AZ UK; 5 Department of Electrical and Computer Engineering, Ajman University, P.O. Box 346, Ajman, United Arab Emirates; 6 Center of Medical and Bio-Allied Health Sciences Research, Ajman, United Arab Emirates; 7 Department of Food Science and Human Nutrition, Laboratory of Food Quality Control and Hygiene, Agricultural University of Athens, IeraOdos 75, Athens, 11855 Greece; 8 Department of Physics, University of Patras, 26504 Patras, Greece

**Keywords:** Materials science, Nanoscience and technology

## Abstract

Although organic light-emitting diodes (OLEDs) are considered a mature technology, further enhancements in their efficiency are of paramount importance for advancing their incorporation in high-quality displays and flexible, wearable, electronic devices. In this regard, we propose an innovative approach, focusing on strategic modifications to the hole transport layer (HTL) through the integration of core-shell nanoparticles. Silver nanoparticles (Ag-NPs) encapsulated in a tungsten polyoxometalate compound (POM) are embedded within the prototype poly(3,4-ethylenedioxythiophene)-poly(styrenesulphonate) (PEDOT:PSS) to form the modified HTL. Our work reveals the pivotal plasmonic role of Ag-NPs in enhancing OLED device performance based on commercially available conjugated polymers. Comprehensive analyses, including UV–Vis absorption spectroscopy, atomic force microscopy, photoluminescence spectroscopy, and electrical measurements, confirm the influence of the POM encapsulated Ag-NPs on improving the device efficiency. This is attributed to the synergistic influence of enhanced hole injection and conductivity and beneficial optical effects (i.e. the Localized Surface Plasmon Resonance (LSPR) and, likely, light scattering of the POM-Ag NPs in the core-shell configuration, depending on their diameter), contributing to enhanced carrier balance and exciton recombination rate. Comparison with POM gold NPs (POM-Au NPs) highlights the distinct advantages of POM-Ag NPs. Our work reveals the potential of this innovative approach to contribute to the evolution of high-performance OLEDs, ensuring a visually compelling and efficient future.

**Supplementary Information:**

The online version contains supplementary material available at 10.1038/s41598-024-79977-w.

## Introduction

In recent decades, the revolutionary discovery of Organic Light-Emitting Diodes (OLEDs) has pioneered next-generation technologies based on organic optoelectronics, including digital displays, solid-state lighting, sensors and flexible electronics^1^. OLEDs are very appealing due to their exceptional electro-optical characteristics that effectively satisfy the evolving needs of various industries combined with cost-effective manufacturing processes. As a result, displays with superior contrast ratios, broad viewing angles and low power consumption that are highly efficient, lightweight, thin, and flexible have been demonstrated by industrial display manufacturers^2^. However, to maximize the commercial potential of OLED technology, two pivotal challenges must be addressed: enhancing efficiency and reducing production costs^3^. A synergistic effort between academia and industry to explore novel materials and develop more efficient device structures will enhance the performance of OLEDs, extend their lifespan, and further reduce power consumption^4^.

A significant percentage of photons generated inside an OLED device become trapped within the structure, leading to a decrease in the external electroluminescence quantum efficiency (EQE)^5^. Enhancing the efficiency and overall performance of OLEDs relies on increasing their internal electroluminescence quantum efficiency (IQE)^6^. Consequently, extensive research has focused on the multilayer OLED architecture with the addition of appropriate charge transport layers. The significance of both the electron transport layer (ETL) and the hole transport layer (HTL) is closely tied to their roles in regulating charge transfer from the respective electrodes (cathode and anode) to the emissive layer (EML). Hence, these layers enable the efficient injection and transport of charge carriers into the EML, contributing to effective radiative recombination and photon generation^7^.

In OLEDs, HTLs play a pivotal role in mitigating exciton quenching at the anode/EML interface and enhancing hole injection upon optimizing the interfacial energy alignment^8^.In particular, suitable HTLs ensure increased and efficient hole injection, resulting in an increase in the radiative electron-hole recombination rate and a reduction of the device’s operating voltage. Additionally, they act as buffer layers, preventing undesired electron migration from the EML to the anode and, thus, enhancing device performance^9^. To achieve these goals, HTL materials are characterized by a high work function with a low-lying Highest Occupied Molecular Orbital (HOMO) and a high-lying Lowest Unoccupied Molecular Orbital (LUMO)^10^ for optimal interfacial energy alignment between the anode and the EML, reducing the energy barrier for hole injection into the EML and enhancing charge balance and carrier recombination in the EML^11,12^.

A well-established HTL material, PEDOT:PSS, is a p-type conjugated polymer in aqueous solution known for its exceptional properties, including high electrical conductivity and workfunction, excellent film-forming properties, transparency to visible light in its doped state, and being a low-cost material in addition to its compatibility with other organic or inorganic materials^12^. To further improve its optoelectronic and morphological properties (in particular its conductivity and workfunction) and develop a more robust, water-processable formulation, various research groups have integrated metal NPs into PEDOT: PSS^13–16^. This integration combines the conductive polymer benefits of PEDOT: PSS with the unique plasmonic properties of metal NPs. Metal or other inorganic such as metal oxide NPs have also been incorporated in various fluorescent and phosphorescent OLEDs not only in the HTL but also in different positions in the device architecture such as the HTL/EML interface or within the EML and have demonstrated their positive influence on device efficiency and stability^17–21^. Notably, enhanced OLED performance has been attributed among other reasons to harnessing the strong, tunable, Localized Surface Plasmon Resonance (LSPR) effect of the metal NPs^22^.

**Fig. 1:**
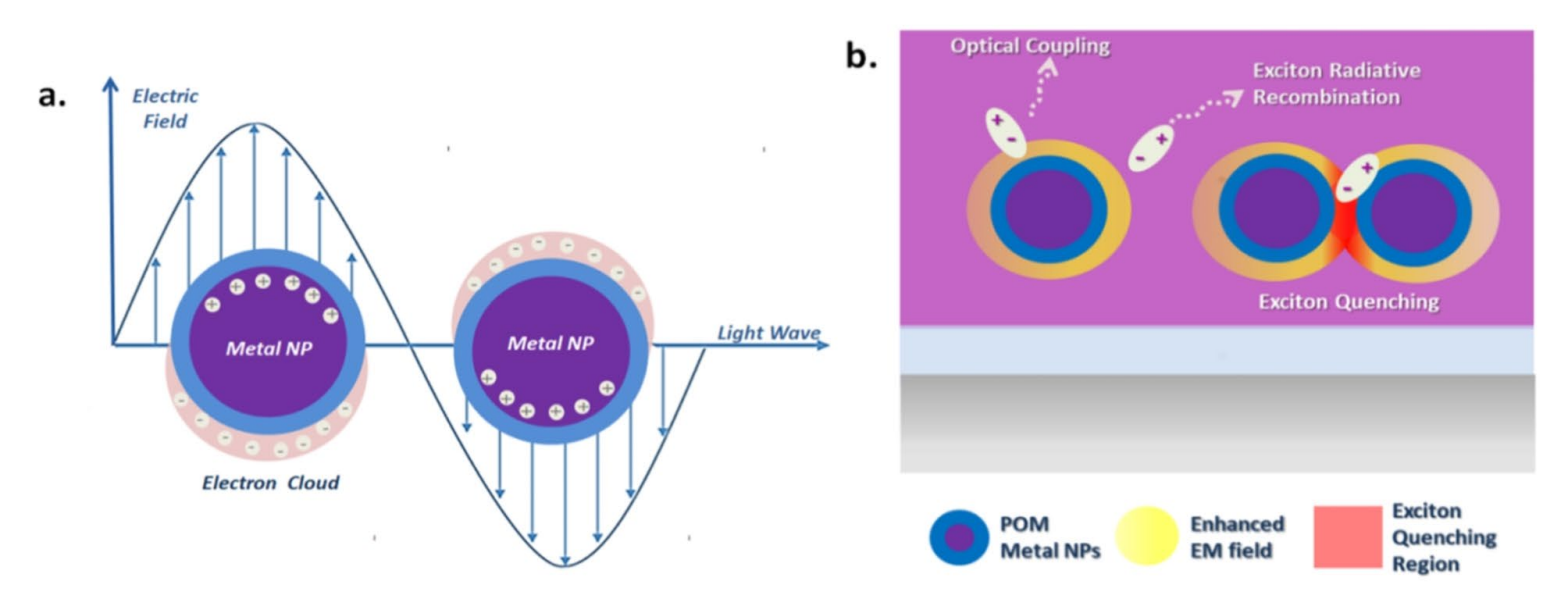
(**a**) “Electron Cloud” States in Core-Shell Ag-NPs induced by interaction with incident electric field, (**b**) Radiative and non-radiative interactions between Ag-NPs and excitons due to LSPR phenomenon.

During the LSPR phenomenon, the incident electromagnetic (EM) field, generated by the radiative recombination of excitons within the EML, interacts with metallic NPs^23,24^. This interaction is particularly notable in noble metals such as silver (Ag), gold (Au), and copper (Cu) due to their unique electronic properties. The oscillating electric field of the incident light induces coherent oscillations of the conduction band electrons within these NPs. When the frequency of the incident light matches the resonance frequency of the surface electrons in the NPs, a condition known as plasmon resonance is achieved. Plasmon resonance leads to the excitation of surface plasmons, which are collective oscillations of the conduction electrons at the nanoparticle’s surface. The resonance condition occurs because the incident EM field exerts a force on the conduction electrons, causing them to oscillate. This resonance excites plasmons on the surface of the NPs and results in a distribution of free surface electrons, forming an ‘electron cloud’ around the NPs^25,26^. Consequently, this ‘electron cloud’ oscillates relative to its original position due to the restoring Coulomb attractive force between the electrons and the nucleus^27,28^. Figure 1a illustrates the “electron cloud” states in core-shell Ag-NPs induced by interaction with an incident electric field.

As a result, the radiative LSPR mechanisms are categorized into two main types, the near-field and the far-field LSPR phenomena^29–31^. The near-field LSPR phenomenon involves the relaxation of incident photons and the re-emission of absorbed light, resulting in the amplification of local electric fields^32^.This refers to the optical coupling between the NPs and incident photons, as the EM field is generated in the immediate vicinity of the metal NPs. On the other hand, the far-field LSPR phenomenon is associated with the scattering of the incident photons into the surrounding area at distances larger than the dimensions of the NPs^33^. These EM waves change the propagation direction when they encounter a NP, which acts as an obstacle along the path of light transmission^34,35^. The LSPR radiative processes strongly depend on the shape, size, interparticle distance, and spacing of metal NPs from the generated excitons^36^. If the metal NPs are too close, either due to their size, high concentration, or if their distance from excitons is too small, there is a potential risk of excitons being susceptible to non-radiative quenching at the surface of metal NPs^37,38^. These phenomena are depicted in Fig. 1b. Generally, spectral matching between the absorption peak of metal NPs and the PL peak of an emitter and their associated overlap increases the likelyhood for resonance between the generated light in the emitter and the LSPR excited by the metal NPs and, thus, may enhance the emission intensity.

Due to the unique electronic structure and dielectric properties of Ag-NPs, they exhibit the most pronounced field enhancement and a strong LSPR effect in the visible and near-infrared regions, as predicted by Mie theory^39,40^. To this extent, our work explores the integration of Ag-NPs into the highly conductive polymer PEDOT: PSS to form a modified HIL/HTL that is then incorporated in OLEDs. It focuses on their impact both as a material and as a HTL in OLEDs that emit in the visible (in particular, green-yellow and blue light) area of the electromagnetic spectrum. Specifically, we first examine the incorporation of 40 nm and 80 nm diameter Ag-NPs into PEDOT: PSS at varying concentrations and study their influence on OLED performance. Electrical measurements of OLEDs based on Ag-NPs modified PEDOT: PSS HTL reveal the absence of luminous efficiency (LE) roll-off at high luminance levels. However, significant degradation of electrical properties is noted, attributed to NP aggregation, which increases non-radiative recombination and leads to exciton quenching. Notably, larger sizes or higher concentrations of Ag-NPs lead to aggregate formation due to strong electrostatic forces.This degradation mechanism underscores the importance of effective encapsulation of Ag-NPs with an appropriate protective dielectric shell.

A core-shell NPs structure will lead to suppression of Ag NP aggregate formation as the distance between NPs will increase with the core acting as a physical separation barrier. Thus, this shell will protect excitons from potential quenching by NPs’ clusters and will retain plasmonic properties of the Ag-NPs avoiding potential environmental degradation or oxidation^41^. An important step toward addressing these challenges is proposed by fabricating a novel HTL, comprising plasmonic core-shell Ag NPs embedded into PEDOT: PSS with an innovative shell made of water-soluble inorganic molecular oxides, derived from polyoxometalate tungsten compounds (POMs). POMs are an attractive option as shells due to their favorable optoelectronic properties such as their high electrical conductivity and tunable energy levels, their photochemical stability upon reduction and their capability to stabilize the metal NPs in nanometer dimensions, suppressing passive mass exchange and charge transfer between the core-metal NPs and its environment^42^.

POM-Ag-NPs were found to exhibit excellent dispersion and compatibility with PEDOT: PSS, maintaining its appealing morphological and optoelectronic properties without significant changes. Furthermore, upon considering the critical role of POMs in protecting the optimal plasmonic properties of Ag-NPs, highly efficient OLEDs based on POM-Ag NPs modified PEDOT: PSS HIL/HTL are demonstrated to be promising candidates for display applications as the core-shell structure synergistically contributes to their enhanced optoelectronic properties. Finally, comparison with POM encapsulated gold NPs (POM-Au NPs) embedded in PEDOT: PSS revealed that OLEDs with POM-Ag NPs modified PEDOT: PSS had a greater potential for LSPR enhancement, as expected from Mie Theory, and achieved higher EQEs.

When Ag-NPs and POM-Ag NPs are integrated into OLEDs, the overlap of the F8BT emission spectrum with the broadened plasmonic absorbance of Ag-NPs extending up to 500 nm might play a significant role. The normalized electroluminescence (EL) spectra of these OLEDs show that the peak emission of F8BT at 532 nm remains almost identical across various volume ratios. Effective optical coupling between the broadened LSPR Ag-NPs peak and the F8BT emission might enhance the local electromagnetic field, facilitate effective energy transfer, and boost radiative recombination rate, thus leading to an enhanced emission intensity. The synergy between these two active components is expected to enhance the optoelectronic properties of the modified OLEDs. Encapsulating Ag-NPs with POMs is identified as an appealing and promising strategy to overcome the limitations of PEDOT:PSS, creating an effective HTL that not only facilitates hole injection and improves carrier balance and recombination but also contributes to an enhanced light emission upon exploring the LSPR phenomenon and reducing exciton quenching at the modified anode/EML interface in the presence of the POM shell. The broadening of the plasmonic peak is proposed to be essential for facilitating the resonance interaction that leads to improved OLED performance with the selected materials.

## Results

### Impact of Ag NPs embedded in PEDOT: PSS as HTLs in OLEDs

**Fig. 2:**
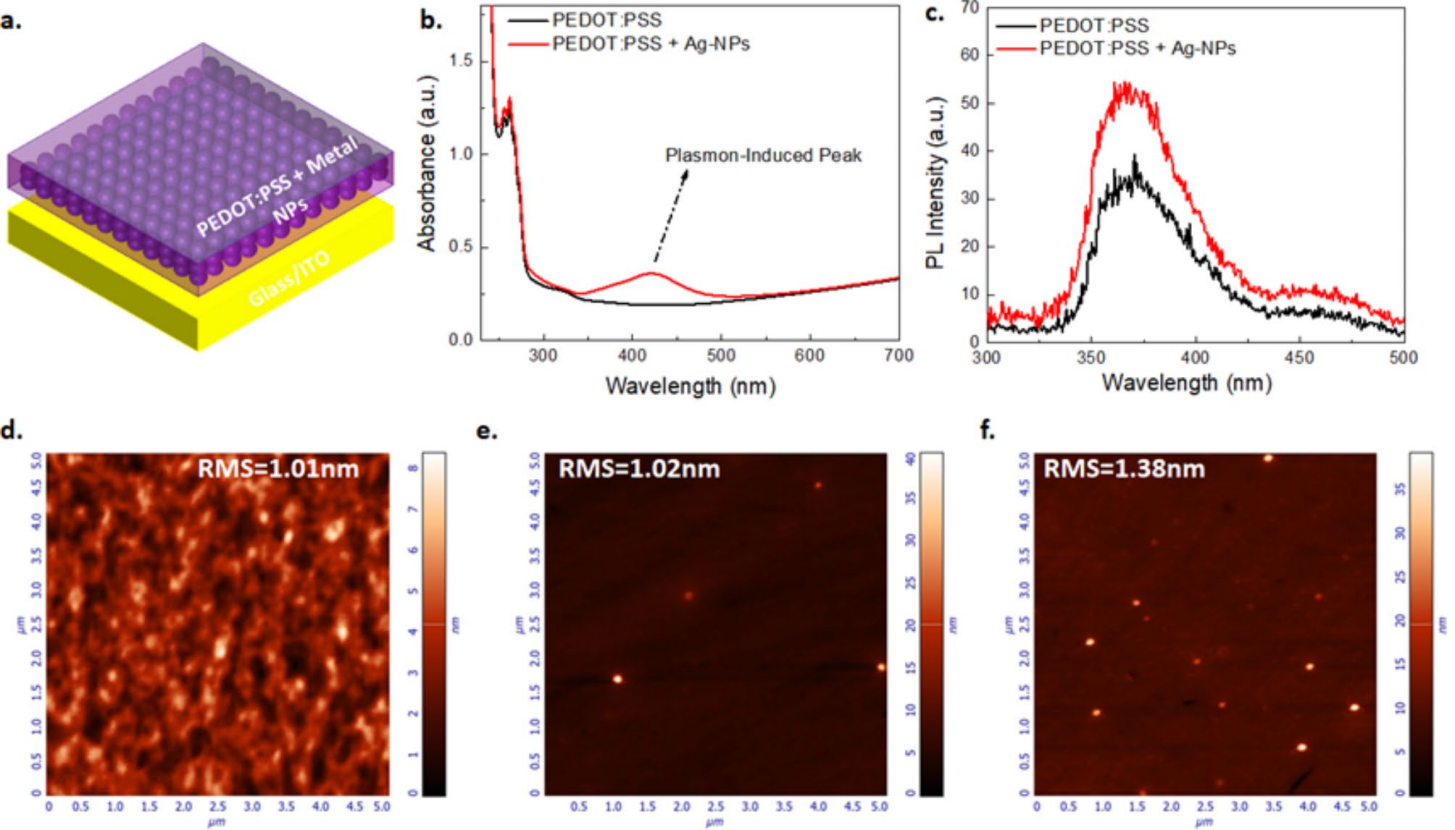
(**a**) Schematic illustration of the interface ITO/PEDOT: PSS + 40 nm Ag-NPs. Ag-NPs were first dispersed into the PEDOT: PSS solution and then the mixed solution was spin-coated on glass/ITO substrate. (**b**) UV-Vis absorption spectra of the PEDOT: PSS and PEDOT: PSS + 40 nm-Ag-NPs solutions. (**c**) Steady-state PL spectra of PEDOT: PSS and PEDOT: PSS + 40 nm Ag-NPs films coated on glass substrates at an excitation wavelength of 320 nm.5 × 5 μm^2^ 2D surface AFM images of the (**d**) PEDOT: PSS film, and (**e**, **f**) PEDOT: PSS + 40 nm Ag-NPs films at 1:1 and 1:4 volume ratios, respectively.

Among various metal NPs, Ag-NPs are distinguished for their UV-Vis light absorption as a result of surface plasmon resonance (with a SPR peak at around 430 nm in an aqueous medium), thus being ideal for organic optoelectronic devices with minimal light loss requirements^43^. Their significant scientific interest lies in their exceptional electrical and optical properties, which are highly dependent on their size, shape and distribution in nanostructures^44^. In this study, commercially available Ag-NPs were incorporated into PEDOT: PSS and spin-coated onto glass-ITO substrates as depicted in Fig. 2a to explore their optoelectronic and morphological properties and then evaluate their role when they are employed as the HTL in OLEDs.

The UV-Vis absorption spectra for solutions of PEDOT: PSS and the mixture of PEDOT: PSS with 40 nm Ag-NPs at a concentration of 10^− 4^ M are presented in Fig. 2b. These spectra display several absorption peaks characteristic of the individual components, indicating that integrating Ag-NPs with PEDOT: PSS does not change the electronic structure of PEDOT: PSS and that no ground state electronic interaction occurs between PEDOT: PSS and Ag-NPs. The peak at around 260 nm is due to the π-n transition (bonding to nonbonding) in the aromatic ring of PSS (the corresponding π-π*, bonding to antibonding, transition of the aromatic rings shows a peak at 223 nm, not shown here)^16^. Moreover, the wide absorption band occurring between 500 and 700 nm is due to the neutral state of PEDOT (note that polaron or bipolaron bands appear in the NIR region)^45,46^. The lack of any additional peaks in the PEDOT: PSS spectrum confirms the polymer’s purity. On the other hand, in the spectrum of the PEDOT: PSS + Ag-NPs mixture, a new characteristic, relatively broad, peak at 422 nm appears, which is indicative of the plasmonic induced effect of Ag-NPs (i.e. the Ag-NPs LSPR peak). The width (FWHM, full-width half-maxima) of the LSPR is estimated to be around 74 nm. According to Mie theory, spherical NPs exhibit a single, sharp, plasmonic–induced peak, whereas other non-spherical shapes, like nanorods, show multiple peaks due to various resonance modes^47,48^. Although the peak at 422 nm is not as sharply defined as expected for spherical NPs, the absence of characteristic multiple peaks suggests that the NPs are mostly spherical. Also, this wide plasmonic-induced peak indeed corresponds to Ag-NPs with diameters ranging from 10 to 100 nm as it is located in the range expected for that NP size, namely between 380 and 500 nm^49,50^.

The optical properties of these materials were further investigated using steady-state photoluminescence (PL) spectroscopy, as shown in Fig. 2c, where the excitation wavelength was set at 320 nm. Both the pristine PEDOT: PSS and the PEDOT: PSS with 40 nm Ag-NPs films exhibit broad emission spectra with the PL peak located at 369 nm. This indicates that the incorporation of Ag-NPs into PEDOT: PSS does not alter the excited state emission. However, the slightly enhanced PL emission intensity observed in the Ag-NPs-modified PEDOT: PSS films may reasonably assume to originate either from the aggregation of Ag-NPs in the polymer matrix providing light scattering centers or from the spectral overlap between the Ag NP LSPR peak and the PEDOT: PSS PL spectrum, a requirement noted above for effective energy transfer and enhanced light emission.

A 2D topological analysis was conducted using Atomic Force Microscopy (AFM) to gain insight into the spatial distribution of 40 nm diameter Ag-NPs in the surface of PEDOT: PSS at varying concentrations and different diameters and their influence of film RMS roughness (refer to Fig. 2(d-f) and Figure S3(e-f)). The AFM measurements of the PEDOT: PSS films modified with 40 nm Ag-NPs reveal that higher concentrations of NPs result in increased RMS roughness values due to increased NP aggregation, as shown in Fig. 2f. Specifically, the surface of PEDOT: PSS is smooth, with a root mean square (RMS) roughness of 1.01 nm, while with the incorporation of Ag-NPs into PEDOT: PSS at a volume ratio of 1:1, the RMS roughness is very similar (1.02 nm). However, in a PEDOT: PSS film with Ag-NPs at a higher volume ratio of 1:10, aggregation of Ag-NPs is observed, resulting in an increased RMS roughness of 1.38 nm.NP aggregation is suggested to elevate the film surface roughness and is mainly attributed to a greater number of collisions between Ag-NPs, leading to either attachment or repulsion^51,52^. In addition to repulsive and attractive interactions among NPs, Van der Waals forces also play a role, exhibiting a inverse six power dependence on interparticle distances^53,54^. Furthermore, the diameter of the NPs significantly impacts this phenomenon, where larger diameters lead to reduced interparticle distances, as demonstrated in Figure S3f, which features PEDOT: PSS embedded with 100 nm diameter Ag-NPs.

In contrast, Fig. 2e demonstrates that those 40 nm diameter Ag-NPs are well-dispersed into PEDOT: PSS, without noticeable aggregation, minimally affecting surface RMS roughness. Thisis attributed to the water-soluble nature of the NPs, which facilitates the creation of smooth, thin films. Consequently, AFM images show that films integrating 40 nm diameter Ag-NPs into PEDOT: PSS at a 1:1 volume ratio exhibit reduced self-assembly of NPs, suggesting their suitability as an optimal HTL material. The absence of any distinct Ag-NPs aggregation minimizes non-radiative recombination of electron-hole pairs and does not negatively affect LSPR.

**Fig. 3:**
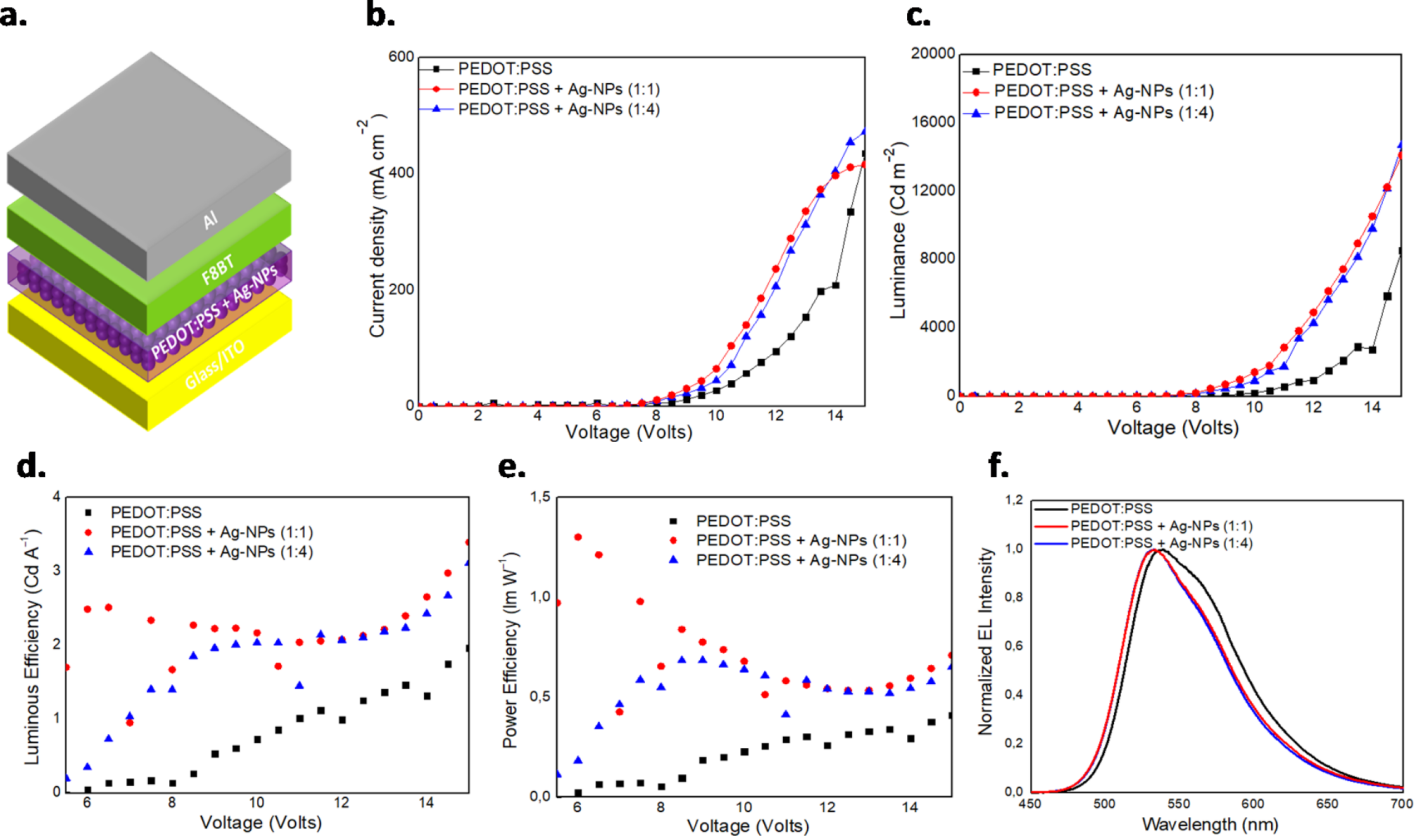
(**a**) Schematic representation of the OLED device architecture. (**b**) Current density - Voltage and (**c**) Luminance – Voltage characteristic curves of the fabricated OLEDs based on as-deposited PEDOT: PSS and PEDOT: PSS + Ag-NPs at different volume ratios. (**d**-**e**) Luminous and Power Efficiency versus Voltage (LE-V and PE-V), respectively. (**f**) Normalized EL spectra of the same OLEDs at a voltage of 17.5 V. The size of the NPs used in these devices was 40 nm.

To comprehensively evaluate the impact of incorporating Ag-NPs with diameters of 40 nm and 80 nm into PEDOT: PSS HTLs, modified OLEDs were developed. The precise OLED structure, depicted in Fig. 3a, consists of indium-tin oxide (ITO) coated glass substrates for the transparent anode, Ag-NPs embedded in PEDOT: PSS as the HTL, the F8BT (poly[(9,9-dioctylfluorenyl-2,7-diyl)-alt-co-(1,4-benzo-{2,1,3}-thiadiazole)]) green-yellow polymer for the EML, and aluminum (Al) for the cathode. Additionally, a reference device without Ag-NPs was used for comparison. In Fig. 3(b-f) and Figure S1, current density-voltage (J-V) and luminance-voltage (L-V) characteristics were examined to investigate hole injection and transport towards the F8BT EML for both 40 nm and 80 nm NPs diameters, respectively. The findings reveal an enhanced device performance by integrating Ag-NPs into PEDOT: PSS at various concentrations. This incorporation of Ag-NPs into PEDOT: PSS leads to increased current density and luminance at the same voltage, compared with the reference OLEDs. These improvements may be attributed to the enhanced hole injection and transport in the Ag-NP modified OLEDs, leading to better charge balance and a higher exciton recombination rate, as also supported by transient-resolved PL (TRPL) measurements presented in Figure S2. The highest performance for PEDOT: PSS + Ag NPs modified OLEDs was achieved at concentration ratios of 1:4 and 1:2 for 40 nm and 80 nm Ag NPs, respectively. The devices with different diameters of Ag NPs demonstrated the same maximum current density and luminance values, at 733 mA/cm² and 18.750 Cd/m², respectively, surpassing the corresponding values of the reference device (240 mA/cm² and 9.059 Cd/m²).

OLEDs with a concentration ratio of 1:4 for PEDOT: PSS and 40 nm Ag-NPs, exhibited a lower turn-on voltage of 7.28 V (defined at 10 Cd/m²) compared to the reference device. The device with a concentration ratio of 1:1 shows a higher turn-on voltage at 8.92 V, while the reference device registers a turn-on voltage of 11.5 V. On the other hand, OLEDs using 80 nm Ag NPs displayed higher turn-on voltage values with increasing Ag-NP concentrations, surpassing those of the reference device.

In Fig. 3(d-e) and S1, OLEDs based on 40 nm and 80 nm Ag NPs demonstrated the potential to attain higher luminous efficiency (LE) at higher luminance values that surpass the standard reference point of 10^4^ Cd/m^2^. For 40 nm Ag NPs, the maxima LE values surpassed the maxima LE values of the reference devices by a factor of 2 whereas an up to an order of magnitude improvement was demonstrated for lower luminance values for the optimum 1:1 volume ratio. For 80 nm NPs, no significant improvement in the LE values was observed but the maxima LE values but are reached at higher luminance values likely due to the combined effects of moderating carrier balance and radiative recombination induced by the presence of Ag-NPs into PEDOT: PSS. The absence of the roll-off phenomenon indicates that degradation mechanisms, such as exciton–exciton annihilation and exciton–charge annihilation, are mitigated^55^. We also note that the larger diameter Ag-NPs may have a smaller effect in hole injection efficiency due to the LSPR excitation. Furthermore, the normalized EL spectra, presented in Fig. 3f and S1d, show a slight but discernible blue shift in the peak emission wavelength and a small reduction of the FWHM of the Ag-NP modified PEDOT: PSS based OLEDs.All devices featuring 40 nm and 80 nm Ag-NPs consistently exhibit their peak emission at 532 nm, in contrast to the reference device, which shows an EL peak at 538 nm. The observed blueshift may be reasonably ascribed either to the influence of the LSPR phenomenon induced by these NPs with an enhanced recombination rate or to a microcavity effect and a small change in the location of the recombination zone.

To further explore the behavior of Ag-NPs embedded in PEDOT: PSS as a modified HTL in OLEDs, electrical measurements were performed on devices with an EML based on a phenylene based (namely BE120) polymer, which emits blue light instead, as shown in Figure S3(a-d). These devices, incorporating 40 nm and 100 nm Ag-NPs, revealed that the optimal values of current densities and luminance occurred in those with PEDOT: PSS and 40 nm Ag-NPs at a volume ratio of 1:1, with corresponding values of 260.66 mA/cm^2^ and 33.14 Cd/m^2^, instead of the reference values of 80.90 mA/cm^2^ and 19.89 Cd/m^2^.However, these devices exhibited a 56% lower maximum LE compared to the reference OLEDs. That observation suggests a lower carrier balance in these devices utilizing both 40 nm and 100 nm Ag-NPs probably as a result of the excess hole injection and/or the unfavorable interfacial energy level alignment with the HOMO of the BE phenylene-based polymer which lies at 5.2 eV (compared to the 5.8 eV in F8BT).

To summarize, OLEDs with Ag-NPs modified PEDOT: PSS as HTL mitigate the roll-off phenomenon and achieve low turn-on voltages, yet optimizing their size is key to leveraging LSPR effects. Larger Ag-NPs aggregate, while smaller ones absorb photons, reducing light scattering. To prevent self-assembly of Ag-NPs driven by electrostatic and Van der Waals forces while, simultaneously, maintaining their plasmonic properties requires effective encapsulation using the core-shell structure principle. Encapsulation with Keggin-type tungsten POM compounds may be a promising approach. These compounds offer uniform encapsulation of Ag NPs, preventing aggregation and preserving their size and morphology while maintaining their plasmonic nature^56^.

### Impact of Embedding Core-Shell POM Ag NPs in PEDOT: PSS

Incorporating a protective shell based on a Keggin structure of a tungsten POM compound, namely H_2_W_12_$\:{\text{O}}_{40}^{6-}$, around Ag-NPs, enhances the chemical compatibility between Ag-NPs and the host material, PEDOT: PSS^41,42^. This occurs because POM molecules are characterized by chemical stability, including resistance to oxidation and thermal decomposition, which could degrade their plasmonic properties and reduce their effectiveness. Thermal decomposition resistance also ensures that POM maintains its structural integrity and protective capabilities under the operating conditions of the OLEDs. Furthermore, the POM shell is expected to protect Ag-NPs from aggregation and potentially improve their performance in applications that rely on their dispersed state^57^. Experiments were conducted using various concentration ratios of core-shell POM-Ag-NPs and PEDOT: PSS to examine their optoelectronic properties and, next, they were incorporated as HTLs in OLEDs as HTLs in OLEDs.

**Fig. 4:**
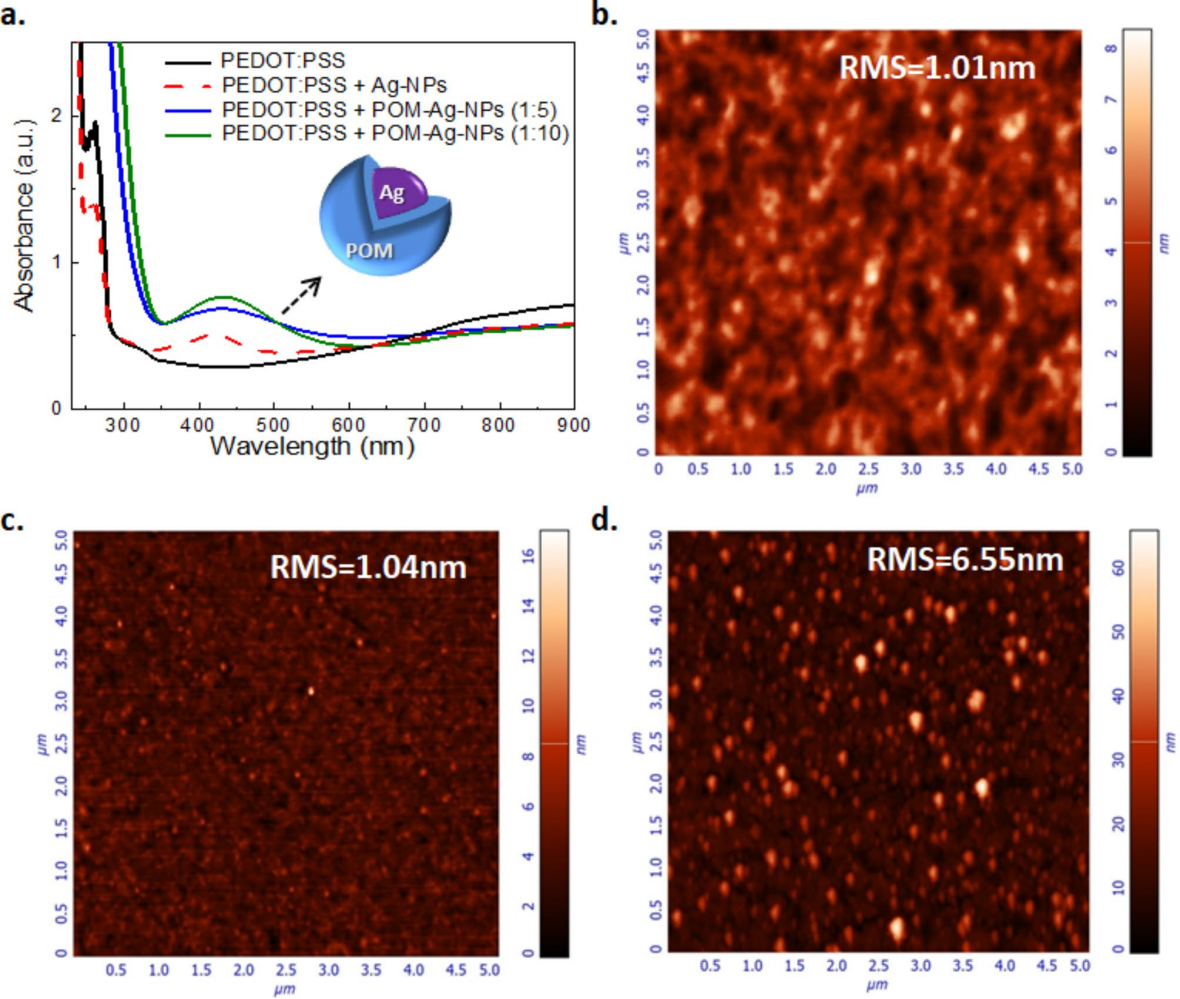
(**a**) UV-Vis spectra of PEDOT: PSS and PEDOT: PSS + POM-Ag NPs solutions at various volume ratios. 5 × 5 μm^2^ AFM surface topographies of (**b**) PEDOT: PSS film, (**c**, **d**) PEDOT: PSS + POM-Ag NPs films at 1:1 and 1:10 volume ratios, respectively.

The UV-Vis spectra for PEDOT: PSS and PEDOT: PSS/POM-Ag NPs solutions are presented in Fig. 4(a). These spectra exhibit multiple absorption peaks, demonstrating that both POM-Ag NPs and PEDOT: PSS maintain most of their properties in the mixture. In the solutions of PEDOT: PSS/POM-Ag NPs with volume ratios of 1:5 and 1:10, plasmon-induced absorption peaks are observed at 430 nm and 432 nm, respectively. This indicates that an increase in POM-Ag NPs concentration leads to a minor red-shift in the plasmonic absorption peak toward longer wavelengths, accompanied by an increase in intensity, consistent with the Beer-Lambert law. Additionally, the plasmon resonance peak of POM-Ag NPs is red-shifted compared with that of Ag NPs as it is affected by the introduction of a core-shell structure and the concomitant variations in the dielectric environment surrounding the NPs. It is noted that the LSPR peaks of the PEDOT: PSS + POM-Ag NPs exhibit broader line widths (~ 92 nm for volume ratio of 1:5 and 90 nm for the 1:10) compared with the PEDOT: PSS + Ag NPs without the POM encapsulation (Fig. 2b). Typically, in a colloidal solution, Ag-NPs are surrounded by water, forming a uniform medium that produces a sharper and more distinct localized surface plasmon resonance (LSPR) peak. When Ag-NPs are embedded in an inert matrix like the polymer film PEDOT: PSS and POM, various factors can cause the plasmonic peak to broaden including changes in the local dielectric constant around the NPs, slight aggregation as well as light scattering. Furthermore, interactions between the matrix and the NPs, such as those involving polar groups in PEDOT: PSS, can also influence the broadening of the plasmonic peak. This broad plasmon resonance could yield higher field enhancement leading to improved OLED performance. The spectra also show suppression of the characteristic splitting peaks of PEDOT: PSS at 255 nm and 261 nm, indicating a potential charge transfer phenomenon between tungsten POM and the aromatic ring of PSS^58^. The plasmon resonance peak of metal NPs is intricately linked to their size, where a red-shift can result from size alterations^59^. Moreover, variations in the dielectric environment surrounding the NPs, due to the insertion of a core-shell configuration, play a pivotal role in influencing their plasmonic properties^60^.

AFM measurements of the films of these materials are depicted in Fig. 4(b-d). Initially, the PEDOT: PSS film exhibited an RMS roughness of 1.02 nm. Incorporating POM-Ag NPs into PEDOT: PSS at a 1:1 volume ratio did not result in any noticeable alteration to the surface of PEDOT: PSS. It is evident that NP aggregation is inhibited and the core-shell NPs are well-dispersed within PEDOT: PSS, likely due to the encapsulation and stabilization effect of POM on the Ag-NPs. However, higher concentrations of Ag NPs led to a substantial increase in RMS roughness, reaching approximately six times higher. Specifically, at a concentration of 1:10 volume ratio, this roughness significantly increased to 6.55 nm due to the much higher concentration of POMs compared to PEDOT: PSS. Higher surface roughness generally contributes to an expanded interface area with the active layer, facilitating enhanced hole injection and an increase in the LSPR phenomenon. Nevertheless, excessively rough surfaces may slow down charge transport, generate additional energy disorder and facilitate non-radiative exciton recombination, thus negatively impacting OLED device performance by causing efficiency degradation.

Additionally, TEM images in Figure S4 offer an analysis of the distribution, size, and shape of the POM-Ag NPs. More specifically, Figure S4a, set at a 100 nm scale, reveals mostly spherical NPs with diameters ranging from 50 to 60 nm. These NPs are well-dispersed, although there is minor aggregation, likely resulting from high interparticle interactions. Furthermore, Figure S4b, at a 200 nm scale, provides a broader perspective, confirming a good overall distribution with slight clustering. This larger scale view emphasizes the homogeneity of the dispersion and provides detailed insights into the extent of aggregation and spatial distribution within the sample.

### Embedded Core-Shell NPs in PEDOT: PSS at the interface with F8BT

The undesirable phenomenon of exciton quenching in the vicinity of metals such as Ag NPs may negatively impact light emission efficiency in OLEDs by causing the energy to be lost through non-radiative processes instead of being transformed into photons. Thus, POMs used as the protective shells to Ag-NPs, upon forming a Ag NP core-POM shell configuration, were alternatively incorporated into PEDOT: PSS to examine their effects in its optical and morphological properties. This approach is expected to mitigate exciton quenching and Ag-NP aggregation which are detrimental to OLED performance. Initially, steady-state PL measurements were performed in ITO/PEDOT: PSS + POM-Ag NPs/F8BT films at 1:10 and 1:2 volume ratios to evaluate how integrating POM-Ag NPs into PEDOT, used as HTL, influences the emission characteristics of F8BT-based OLED structures. In addition to the reference film of ITO/PEDOT: PSS/F8BT, PL measurements were conducted for other films such as ITO/PEDOT: PSS + POM/F8BT and ITO/PEDOT: PSS + Ag-NPs/F8BT at volume ratios 1:10 and 1:2 aiming to separately investigate the influence of POMs and Ag NPs on F8BT light emission.

**Fig. 5:**
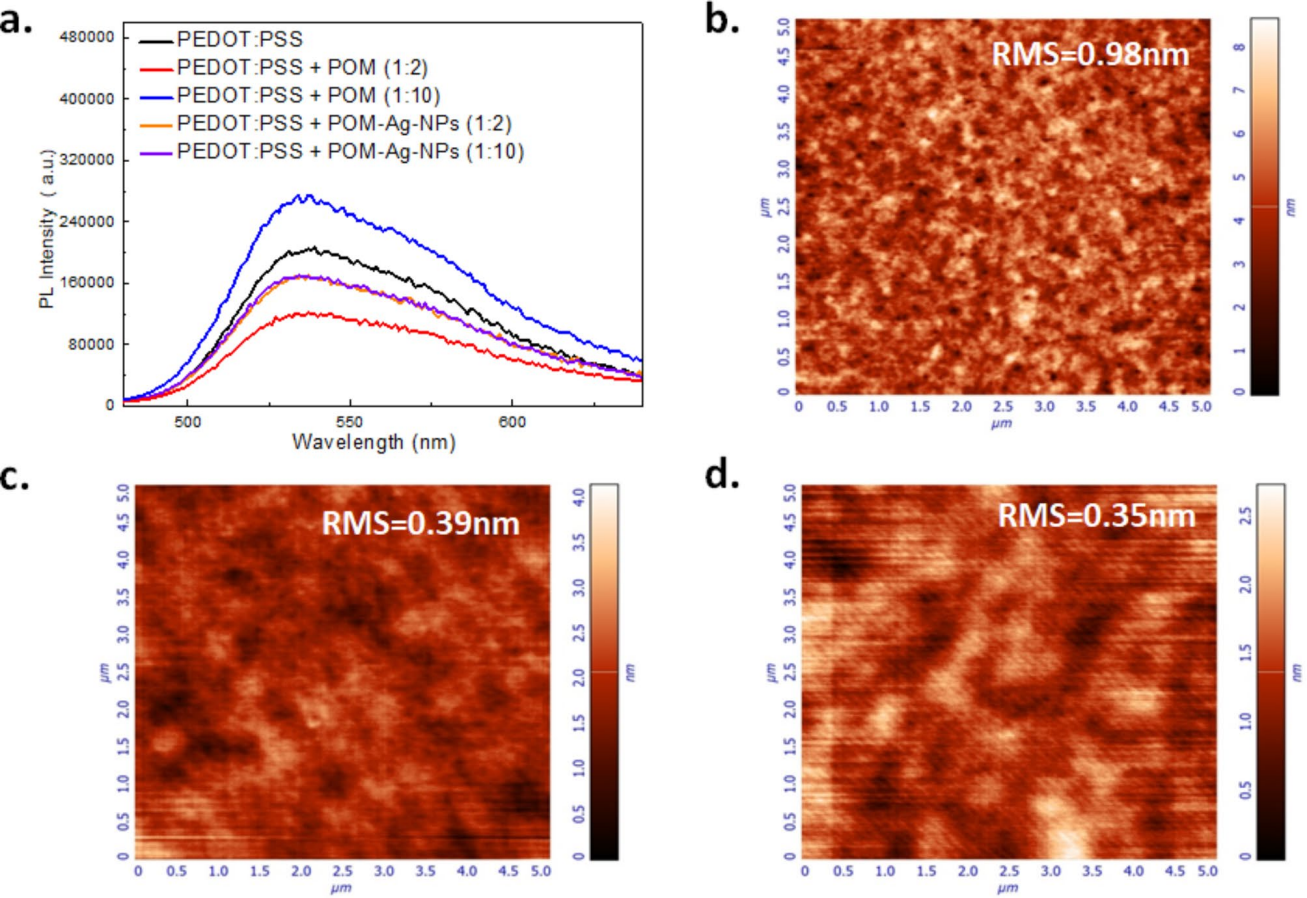
(**a**) Steady-state PL spectra of pristine ITO/PEDOT: PSS/F8BT, ITO/PEDOT: PSS + POM/F8BT, ITO/PEDOT: PSS + 40 nm Ag-NPs/F8BT and ITO/PEDOT: PSS + POM-Ag NPs/F8BT films at 1:2 and 1:10 volume ratios at glass substrates with an excitation wavelength of 470 nm. 5 × 5 μm^2^ AFM images of F8BT spin-coated on (**b**) PEDOT: PSS, (**c**) PEDOT: PSS + POM-Ag NPs (1:2), and (**d**) PEDOT: PSS + POM-Ag NPs (1:10).

In Fig. 5a, which show the PL spectra of these films, it’s evident that tungsten POM and Ag NPs do not modify the peak emission wavelength and the FWHM. Note that films were excited at 470 nm where F8BT strongly absorbs but the selected excitation wavelength is also resonant with the spectral range of the Ag NPs LSPR, resulting in a minor modification (i.e. decrease) of the PL intensity at 536 nm. Also, in Figure S2, the transient PL dynamics of the samples from both F8BT and glass sides indicate that the exciton lifetime is not modified, remaining at approximately 2.15 ns which is a typical value for F8BT. This observation suggests that POMs do not significantly modify the optical properties of F8BT interfaced with the core-shell Ag NPs.

The nanomorphology of these interfaces and the surface morphology of the emissive layer deposited atop the HTL were further examined using AFM measurements (Fig. 5(b-d)). AFM imaging reveals that the films containing POM-Ag NPs exhibit a decrease in RMS roughness by over 50% compared to the PEDOT: PSS/F8BT interface. Additionally, films incorporating POM-Ag NPs, owing to their water-soluble nature, demonstrate reduced thickness and enhanced NP dispersion.

### Effect of POM-Ag NPs integration on OLED performance

**Fig. 6:**
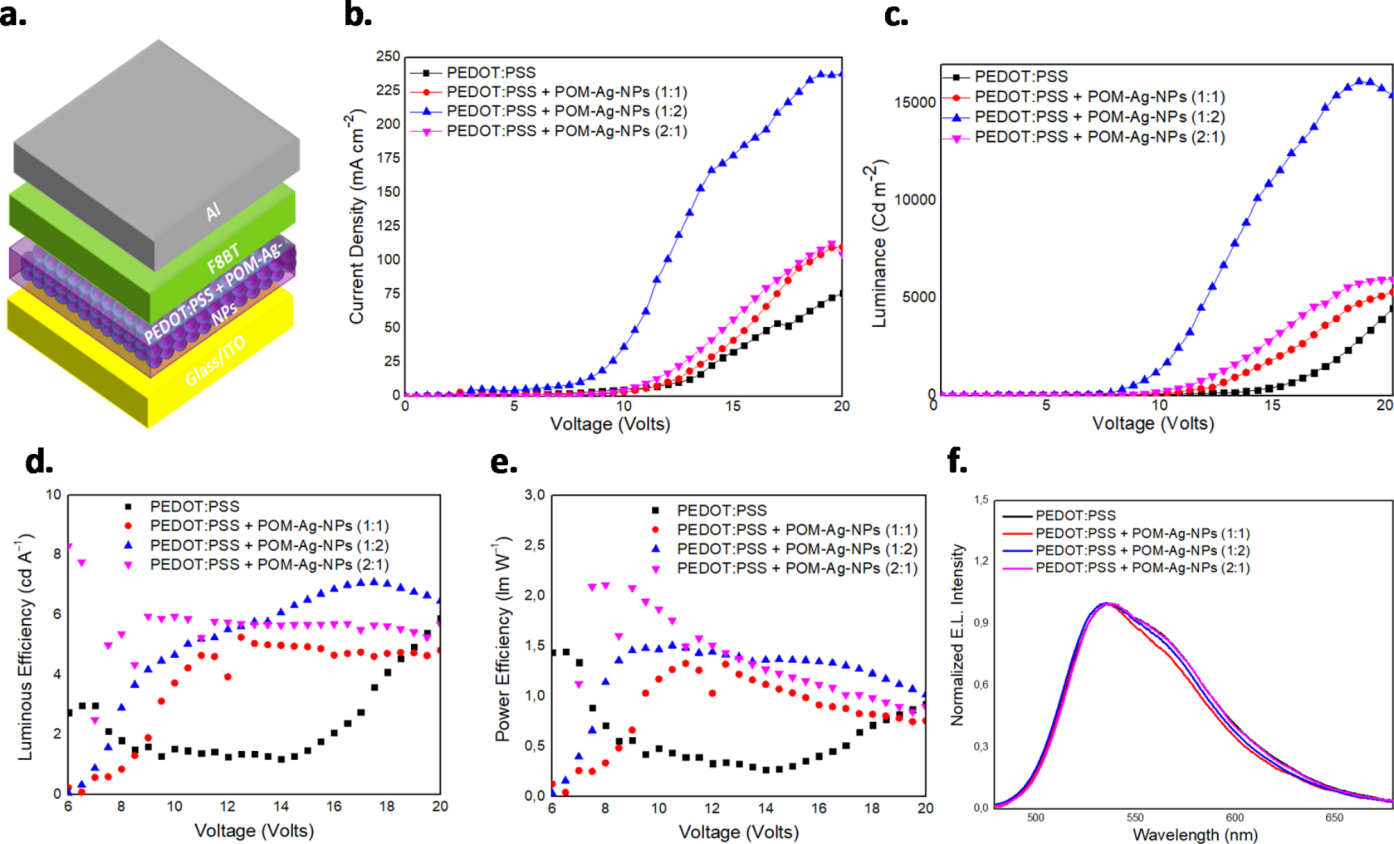
(**a**) Illustration of the OLED architecture, (**b**) Current density-Voltage and (**c**) Luminance-Voltage characteristic curves of the fabricated OLEDs based on PEDOT: PSS and PEDOT: PSS + POM-Ag NPs in various concentrations, (**d**-**e**) Luminous efficiency and Power efficiency vs. Voltage of the same devices and (**f**) Normalized EL spectra at 15 V.

Integrating plasmonic POM-Ag NPs within PEDOT: PSS as an alternative HTL for OLEDs represents a promising approach, with detailed synthesis procedures for POM-Ag NPs in the experimental section. Figure 6a illustrates the detailed architecture of these enhanced OLEDs, showcasing the strategic placement of POM-Ag NPs within the PEDOT: PSS layer to optimize their optoelectronic properties. Subsequent electrical measurements, whose results are depicted in Fig. 6(b-c), reveal significant enhancements in the current density and luminance of the devices as a function of applied voltage, underscoring the pivotal role of POM dielectric coating of Ag-NPs. OLEDs featuring POM-Ag NPs, especially at the optimized concentration ratio of 1:2, demonstrate a notable increase in both current density and luminance. This suggests a decreased hole injection barrier compared to our reference device, indicative of the advantageous influence of POM-Ag NPs on the overall device performance. Specifically, the devices incorporating POM-Ag NPs achieved a peak current density of 238.19 mA/cm² and luminance of 16.113 Cd/m², significantly surpassing the reference device’s values of 75.99 mA/cm² and 4.473 Cd/m², respectively. Moreover, the introduction of POM-Ag NPs resulted in a reduced turn-on voltage from 11.3 V in the reference device to 7.2 V. The lower turn-on voltage can be more likely attributed to the lower hole injection barrier at the modified interface due to the high workfunction of the POM shell as well as the enhanced conductivity of the NP-modified PEDOT: PSS. Smaller improvements are observed in the other two selected concentrations of 1:1 and 2:1. However, the optimal OLED performance appears to show a relatively monotonic trend with increasing POM-Ag NPs concentration up to the optimized 1:2 ratio within PEDOT: PSS (corresponding to the larger POM-Ag NPs loading).

Significant enhancements in LE and PE values are observed and attributed to POM and Ag-NPs’ synergistic effects (Fig. 6(d-e)). Particularly, a strong increase in LE and PE values are seen at low to intermediate luminance values likely due to the considerable improvement in hole injection and transport in the POM-Ag NP modified PEDOT: PSS/F8BT interface. The results highlight the beneficial role of POMs in encapsulating Ag-NPs, as POMs mitigate NP aggregation. Also, the improvement in LE and PE indicates that the shell does not impact the LSPR phenomenon; instead, it may enhance the radiative recombination rate as a result of the progressive improvement in the charge carrier balance with the applied voltage and the likely involvement o light scattering (especially for the larger 80 nm diameter NPs) which enhances light-out coupling and the optical near-field coupling/LPSR effect (for the smaller 40 nm diameter NPs). Moreover, the absence of the roll-off phenomenon at high luminance values, typically associated with the presence of Ag-NPs as potential exciton quenchers particularly at higher populations, underscores their positive influence.

Figure 6f provides insights into the devices’ normalized EL spectra, depicting that the presence of NPs does not exert any significant modification in the emission spectra, which originates from F8BT. Only a slightly smaller FWHM is noted in agreement with our earlier results for the Ag-NP modified HTL. Overall, the introduced NPs regardless of their configuration and properties, have only a minor effect on the OLED emission spectra, highlighting their positive impact on the device’s electro-optical characteristics.

### OLED Performance Comparison: POM-Au NPs and POM-Ag NPs

**Fig. 7:**
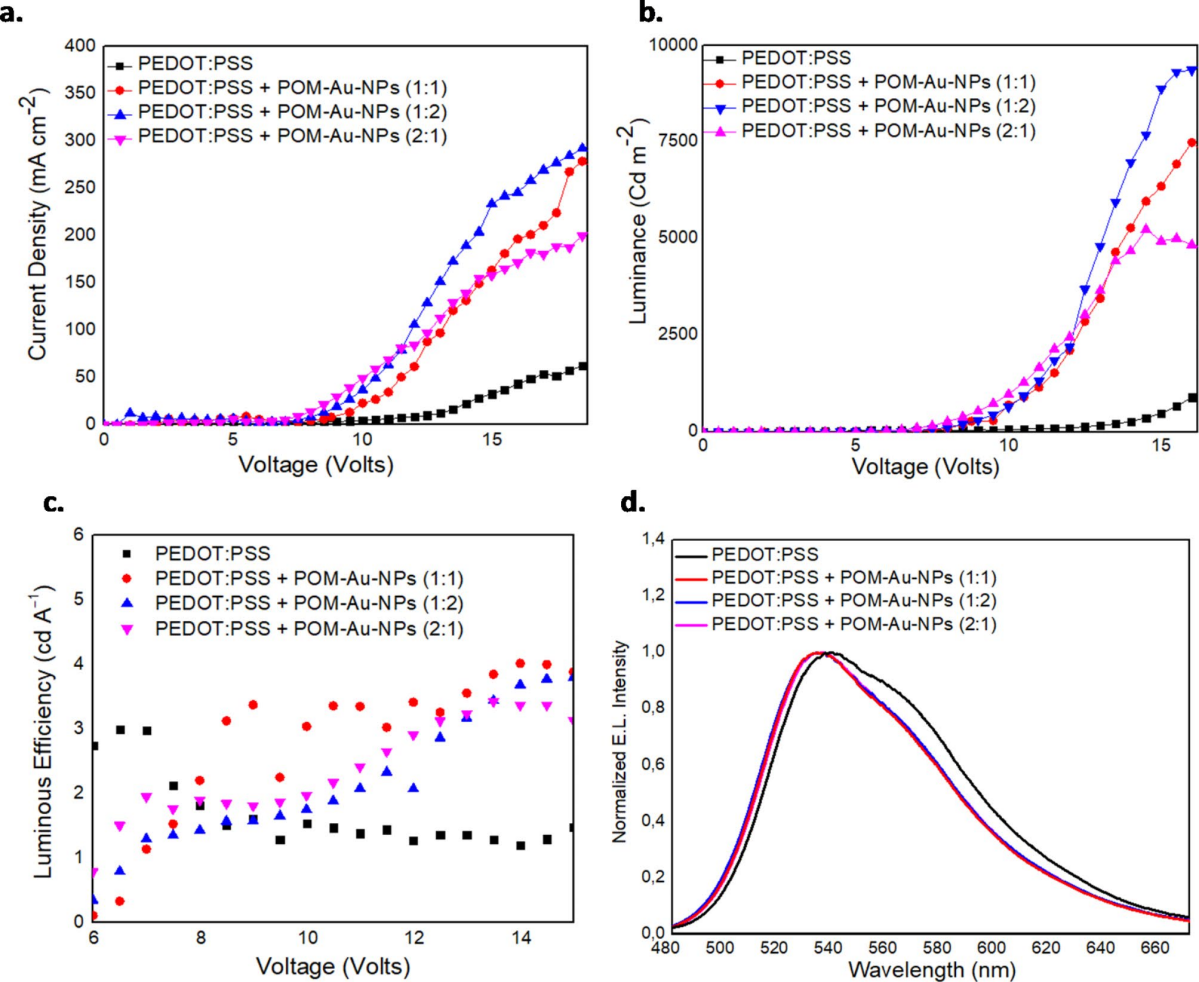
OLED characteristics with PEDOT: PSS + POM-Au NPs (HTL) at 1:1, 1:2, 2:1 volume ratios: (**a**-**b**) Current Density-Voltage and Luminance-Voltage curves, (**c**) Luminous Efficiency-Voltage and (**d**) EL Spectra at 17.5 V.

To gain a deeper insight into the LSPR phenomenon of various metal NPs within PEDOT: PSS in OLEDs, encapsulated gold NPs (POM-Au NPs) were alternatively synthesized and introduced into the PEDOT: PSS layer. In contrast to Ag-NPs, which mainly exhibit far-field broadband LSPR EM enhancement, Au-NPs are known for their stronger near-field EM enhancement^61^. Consequently, electrical measurements in OLEDs incorporating a PEDOT: PSS HTL with embedded POM-Au NPs at various concentrations, demonstrate enhanced optoelectronic properties as presented in Fig. 7(a-d). The highest current density and luminance values were achieved with an HTL based on PEDOT: PSS and POM-Au NPs at a concentration ratio 1:2. Both devices exhibited maximum current density values in the 200–300 mA/cm² range. The POM-Au NPs device demonstrated a maximum luminance of 9407.6 Cd/m², 40% smaller than the equivalent POM-Ag NPs device. Both POM-Ag NPs and POM-Au NPs devices present enhanced luminous efficiencies, with maximum values at 7.1 Cd/A and 4.9 Cd/A, respectively.

POM-Ag NPs based OLEDs demonstrate superior LE values that are maintained at relatively high luminance values compared to those with POM-Au NPs, due to the inherent properties of Ag and their encapsulation within the POM shell that provides advantages in terms of conductivity and superior optical properties. In Fig. 7d, the normalized EL spectrum of POM-Au NPs (embedded in PEDOT: PSS) modified F8BT OLEDs exhibits a peak at 533 nm with a shoulder at approximately 567 nm, which slightly differs from the reference and POM-Ag NPs devices, which emit light at 538 nm. The 5 nm blue-shifted peak of POM-Au NPs may be suggestive of a possible interaction between F8BT excitons and Au-NPs, affecting the radiative recombination mechanism. Overall, modified OLEDs based on POM-Au NPs show enhanced performance, albeit to a lower degree than the POM-Ag NPs, likely due to Ag’s improved LSPR effects and/or the more facile hole injection in a more favorable interfacial energy level alignment. Thus, despite the fact that the LSPR characteristics of the Au NPs may suggest a more favorable spectral overlap with the polymer PL emission compared to the Ag NPs, this was not proven to be the case in appropriately HTL-modified OLEDs suggesting that this resonance interaction may be affected by the presence of the POM shell and/or the NP location in the device structure.

Our work emphasizes the benefits of using POMs to encapsulate Ag or Au-NPs to create core-shell structures that are effectively dispersed in PEDOT: PSS and enhance its properties for potential application as HTLs in OLEDs. Given their excellent physical compatibility with PEDOT: PSS, incorporating POMs with selective metal NPs such as Ag or Au-NPs into PEDOT: PSS is a highly promising strategy for boosting OLED performance.

## Conclusions

In conclusion, this study demonstrates the potential benefits of integrating plasmonic Ag-NPs, coated with tungsten POM compounds, into PEDOT: PSS to employ it as a modified HTL in enhanced performance OLEDs. The encapsulation of Ag-NPs with suitable POM compounds emerges as a pivotal strategy, offering various advantages and significant improvements in optoelectronic devices compared with simple Ag-NPs. Electrical measurements of the POM-Ag NPs modified OLEDs reveal lower turn voltages, increased current densities and luminance values combined with higher efficiencies and suppression of roll-off effects. These improvements in OLED performance are attributed to synergistic effects of the increased hole injection at the modified HTL/EML interface and a likely enhanced conductivity of the POM-Ag NPs modified PEDOT: PSS HTL and the beneficial LSPR effect of the Ag NPs leading to favorable spectral overlap with the polymer PL emission which contribute to enhanced carrier balance and radiative recombination rate. Moreover, the tungsten POM shell protects the functionality of Ag-NPs by preserving their unique optical properties and alleviating their tendency to aggregate in the solid state. Furthermore, a direct comparison of the encapsulated Ag-NPs and Au-NPs reveals that POM-Ag NPs maybe more promising for optoelectronic applications, as they demonstrate enhanced plasmonic effects compared to other metal NPs. This work highlights the impact of appropriately designed metal core – dielectric shell NPs on improving OLED performance by synergistically exploiting metal NP LSPR optical effects and promoting charge/transport at modified interfaces.

## Materials andMethods

### Core-Shell NPs synthesis

Metal core-shell NPs were synthesized via a photochemical method as implemented by Troupis, et al.^62^, wherein the shells from tungsten POMs served multifunctionally as photocatalysts, reducing and stabilizing agents for Ag or Au (core) NP synthesis (referred to as M). In this method, deaerated POM solutions absorb UV light producing excited POM which abstracts electrons from oxidizable organic substrates (S), exemplified by alcohols, producing a blue-colored solution, indicative of the formation of reduced POM (reaction 1). The concentration of the reduced POM can be monitored by visible absorption spectroscopy using its characteristic peak (690 nm for H_2_W_12_O^7 −^ _40_).

1 $${H_2}{W_{12}}O_{40}^{6 - } + {S^{hv}} \to {H_2}{W_{12}}O_{40}^{7 - } + {S_{OX}}$$

The reduced POM obtained by reaction (1) was mixed with a deaerated solution of metal ions (Ag^+^ or Au^III^) and agitated to obtain metal nanoparticles by thermal reaction (2). Upon reacting with metal ions, the characteristic absorbance peak of reduced POM decreases gradually with the simultaneous appearance of the plasmon resonance peak of the metal NPs (430 nm and 532 nm for Ag and Au NPs, respectively).

2 $${H_2}{W_{12}}O_{40}^{7 - } + {M^{N + }} \to {H_2}{W_{12}}O_{40}^{6 - } + M_{_{{\bf{colloidal}}}}^0$$

Metal NPs can be synthesized by the two reactions co-occurring (one-pot system) or separated in time and space (two-pot system)^63^. In a typical experiment, silver nanoparticles (POM-Ag NPs) were synthesized in one-pot system by illumination of a deaerated aqueous solution containing H_2_W_12_O_40_^6−^ (10^− 3^ M), propan-2-ol (1 M), HClO_4_ (0,01 M), and AgNO_3_ (5.7 × 10^− 4^ M) using a 1000 W xenon arc lamp equipped with a cut-off filter (320 nm) to avoid direct photolysis of organic substrate. POM-Au NPs were synthesized in a two-pot system involving first the illumination of a deaerated aqueous solution containing H_2_W_12_O_40_^6−^ (10^− 3^ M) and propanol (1 M) for the production of reduced POM (reaction 1) followed by the addition of a deaerated aqueous solution of HAuCl_4_ (10^− 3^ M) (reaction 2).

### ΟLED devices fabrication process

For the fabrication of OLEDs, indium-tin oxide (ITO) (purchased from Sigma-Aldrich, Athens, Greece) with sheet resistance 15–25 Ω/sq coated on glass substrates was used as the transparent anode. ITO-coated glass substrates were subjected to an ultrasonic cleaning process in sequential baths of deionized water, acetone and isopropyl alcohol (IPA) for 10 min each. Next, the substrates were dried with nitrogen (N_2_) flow and subsequently, they were subjected to a 20-minute UV–Ozone treatment. The PEDOT: PSS (poly(3,4-ethylenedioxythiophene)–poly(styrenesulfonate)) solution with a 1.3 wt% dispersion in H_2_O (purchased from Sigma-Aldrich) was filtered through a polyvinylidene fluoride (PVDF) filter with a pore diameter of 0.45 mm. Filtered PEDOT: PSS solution was spin-coated on the ITO at 6000 rpm for 40 s to serve as the hole injection/transport layer (HIL/HTL). The ITO/PEDOT: PSS films were next placed on a hotplate at 110^o^C for 45 min. For the Ag NP modified PEDOT: PSS HIL/HTL, commercially available Ag-NPs with diameters of 40 nm, 80 nm, and 100 nm solutions were supplied by Alfa Aesar. Solutions were prepared from the mixture of PEDOT: PSS and Ag-NPs, with varying % v/v concentrations. Next, the solutions were also spin-coated onto ITO-coated glass substrates at 6000 rpm for 40 s and annealed on a hotplate at 110 °C for 45 min. F8BT (poly[(9,9-dioctylfluorenyl-2,7-diyl)-alt-co-(1,4-benzo-{2,10,3}-thiadiazole)) and BE120 (Poly[2-(6-cyano-6-methyl-heptyloxy)-1,4-phenylene) were purchased from American Dye Source in Quebec, Canada (ADS 233 YE) and were used as the emissive layers. The green-yellow copolymer F8BT was dissolved in chloroform at a concentration 10 mg mL^− 1^ and after stirring for 2 h at around 40 ^o^C was filtered through a 0.22 μm pore size PTFE filter. Similarly, BE120 was dissolved in chloroform at a concentration of 10 mg mL^− 1^ and the solution was stirred for two hours. Both, F8BT and BE120 solutions were spin coated at 1200 rpm for 40 s to form an approximately 80 nm thin layer and then theywere annealed at 85 °C for 10 min on a hotplate. Finally, OLED device fabrication was completed with the deposition of a 150 nm aluminum (Al) layer as the cathode electrode, through thermal evaporation. The active surface area of each diode was 12.56 mm^2^.

### Characterization techniques

Current density–voltage (J − V) measurements were performed using a Keithley 2400 Power Supply Source-Meter (Vector Technologies, Athens, Greece) in voltage mode with constant incremental steps. Electroluminescence (EL) analysis was executed employing a calibrated photodiode (BPW34 Si PIN photodiode) (Vector Technologies, Athens, Greece). UV–vis absorbance spectra were recorded using a Perkin Elmer Lambda 40 UV–vis spectrometer (Vamvakas-Scientific Equipment, Athens, Greece). Surface morphology was investigated using an NT-MDT AFM system (LaborScience SA, Athens, Greece) in tapping mode. Steady-state PL measurements were carried out on a commercial platform (ARKEO—Cicci Research). Time-resolved PL (TRPL) spectra were measured with an FS5 spectrofluorometer from Edinburgh Instruments (Livingston, UK), utilizing a 478.4 nm laser as the excitation source.

## Electronic supplementary material

Below is the link to the electronic supplementary material.

Supplementary Material 1

## Data Availability

Data is provided within the manuscript or supplementary information files. The datasets used during the current study are also available from the corresponding author on reasonable request.
