# Supplementary material for: Plasmonic enhanced OLED efficiency upon silver-polyoxometalate core-shell nanoparticle integration into the hole injection/transport layer
Source: Sci Rep. 2024 Nov 21;14:28888. doi: 10.1038/s41598-024-79977-w (PMC11582635; doi:10.1038/s41598-024-79977-w)
Supplement: Supplementary file 1 — Supplementary Material 1 [file 41598_2024_79977_MOESM1_ESM.docx]

**Plasmonic Enhanced OLED Efficiency upon Silver-Polyoxometalate Core-Shell Nanoparticle Integration into the Hole Injection/Transport Layer**

**Zoi Georgiopoulou^1,2,#^, Apostolis Verykios^1,#^, Anastasia Soultati^1^, Alexander Chroneos ^3,4,*^, Anastasia Hiskia^1^, Konstantinos Aidinis^5,6^, Panagiotis N. Skandamis^7^, Antonia Gounadaki^7^, Ioannis Karatasios^1^, Theodoros M. Triantis^1^ , Panagiotis Argitis^1^, Leonidas C. Palilis^8,*^, Maria Vasilopoulou^1,*^**

^1^Institute of Nanoscience and Nanotechnology, National Center for Scientific Research ‘Demokritos’,Agia Paraskevi 15310, Athens,Greece.

^2^ Solid State Physics Section, Department of Physics, National and Kapodistrian University of Athens, Panepistimioupolis, Zografos 15784, Athens, Greece.

^3^Department of Electrical and Computer Engineering, University of Thessaly, 38221 Volos, Greece.

^4^Department of Materials, Imperial College, London SW7 2AZ, UK.

^5^Department of Electrical and Computer Engineering, Ajman University, P.O. Box 346, Ajman, United Arab Emirates.

^6^Center of Medical and Bio-Allied Health Sciences Research, Ajman, United Arab Emirates.

^7^ Agricultural University of Athens, Department of Food Science and Human Nutrition, Laboratory of Food Quality Control and Hygiene, Iera Odos 75, 11855, Athens, Greece

^8^ Department of Physics, University of Patras, 26504, Patras, Greece

#These authors contributed equally to this work.

* [alexander.chroneos@imperial.ac.uk](mailto:alexander.chroneos@imperial.ac.uk) (Alexander Chroneos)

^*^ [lpalilis@upatras.gr](mailto:lpalilis@upatras.gr) (Leonidas C. Palilis)

[^*^m.vasilopoulou@inn.demokritos.gr](mailto:*m.vasilopoulou@inn.demokritos.gr) (Maria Vasilopoulou)

**Figure S1.** (a) Current Density - Voltage and (b) Luminance – Voltage characteristic curves of the OLEDs with and without embedded commercial Ag-NPs (80nm diameter) into PEDOT:PSS at volume ratios (1:4), (1:2), (2:1) and (4:1). (c) Luminous efficiency vs. Voltage and (d) EL spectra of the OLEDs at 17.5 V.


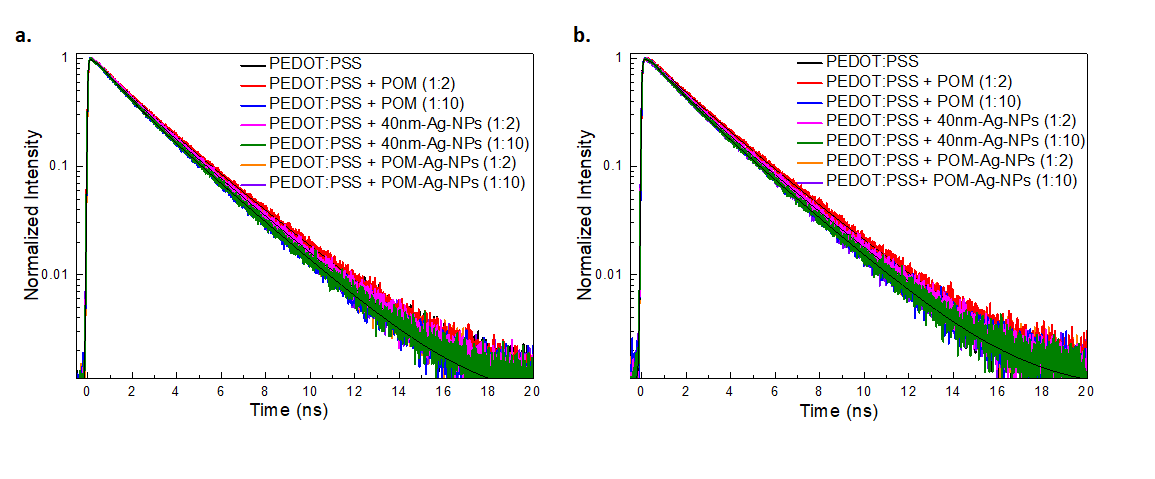


**Figure S2.** Transient PL dynamics of the spectra of ITO/PEDOT:PSS/F8BT, ITO/PEDOT:PSS + POM/F8BT, ITO/PEDOT:PSS + 40nm Ag-NPs/F8BT and ITO/PEDOT:PSS + POM-Ag-NPs/F8BT films at 1:10 and 1:2 volume ratios at glass substrates with excitation wavelength 470 nm (a) with glass side excitation and (b) with F8BT side excitation.

**Figure S3.** (a) Current density - Voltage and (b) Luminance – Voltage characteristics of the OLEDs with and without commercial Ag-NPs with diameters 40nm and 100 nm in PEDOT:PSS at volume ratios 1:1 and 1:4 beneath the emissive layer (BE120). (d-e) Luminous Efficiency vs. Luminance and EL spectra of the OLEDs at a 17.5 V. (e-f) Two-dimensional (2D) height Atomic Force Microscopy (AFM) surface topographies of PEDOT:PSS + 100 nm Ag-NPs films at volume ratios 1:1 and 1:2, respectively. The highlighted region indicates the presence of aggregates.


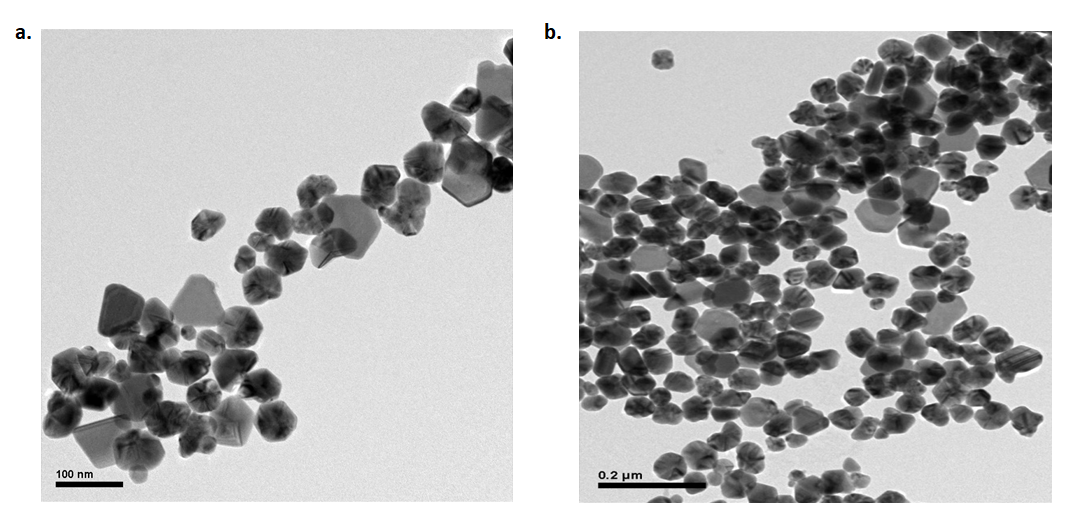


**Figure S4.**Transmission electron microscopy (TEM) images at different scales: (a) 100 nm and (b) 200 nm.
